# Diverged Effects of Piperine on Testicular Development: Stimulating Leydig Cell Development but Inhibiting Spermatogenesis in Rats

**DOI:** 10.3389/fphar.2018.00244

**Published:** 2018-03-28

**Authors:** Xianwu Chen, Fei Ge, Jianpeng Liu, Suhao Bao, Yong Chen, Dongli Li, Yong Li, Tongliang Huang, Xiaofang Chen, Qiqi Zhu, Qingquan Lian, Ren-Shan Ge

**Affiliations:** Department of Anesthesiology, The Second Affiliated Hospital and Yuying Children’s Hospital of Wenzhou Medical University, Wenzhou, China

**Keywords:** piperine, Leydig cell development, rat, cholesterol side chain cleavage enzyme, 3β-hydroxysteroid dehydrogenase, spermatogenesis

## Abstract

**Background:** Piperine is the primary pungent alkaloid isolated from the fruit of black peppercorns. Piperine is used frequently in dietary supplements and traditional medicines. The objective of the present study was to investigate the effects of piperine on the testis development in the pubertal rat.

**Methods:** Piperine (0 or 5 or 10 mg/kg) was gavaged to 35-day-old male Sprague-Dawley rats for 30 days. Serum levels of testosterone (T), luteinizing hormone (LH), and follicle-stimulating hormone (FSH) were measured. The development of adult Leydig cell population was also analyzed 65 days postpartum. For *in vitro* studies, immature Leydig cells were isolated from 35-day-old male rats and treated with 50 μM piperine in the presence of different steroidogenic stimulators/substrates for 24 h.

**Results:** Thirty-day treatment of rats with piperine significantly increased serum T levels without affecting LH concentrations. However, piperine treatment reduced serum FSH levels. Consistent with increase in serum T, piperine increased Leydig cell number, cell size, and multiple steroidogenic pathway proteins, including steroidogenic acute regulatory protein, cholesterol side-chain cleavage enzyme, 3β-hydroxysteroid dehydrogenase 1, 17α-hydroxylase/20-lyase, and steroidogenic factor 1 expression levels. Piperine significantly increased the ratio of phospho-AKT1 (pAKT1)/AKT1, phosphos-AKT2 (pAKT2)/AKT2, and phospho-ERK1/2 (pERK1/2)/ERK1/2 in the testis. Interestingly, piperine inhibited spermatogenesis. Piperine *in vitro* also increased androgen production and stimulated cholesterol side-chain cleavage enzyme and 17α-hydroxylase/20-lyase activities in immature Leydig cells.

**Conclusion:** Piperine stimulates pubertal Leydig cell development by increasing Leydig cell number and promoting its maturation while it inhibits spermatogenesis in the rat. ERK1/2 and AKT pathways may involve in the piperine-mediated stimulation of Leydig cell development.

## Introduction

Piperine (PIP, 1-peperoylpiperidine) is the primary pungent alkaloid isolated from black peppercorns (the fruit bodies of *Piper nigrum*). *Piper nigrum* is a very common food supplement all over the world. Usually, PIP is ingested via diets. After ingestion, PIP is often described as tasteless, soon after which there is a sharp peppery taste.

Piperine has been demonstrated to have many pharmacological activities, including antioxidant properties that can ameliorate the adverse effects of a high-fat diet (Naidu and Thippeswamy, 2002; Vijayakumar et al., 2004). It has also been reported to have many other activities, including anti-diabetic (Atal et al., 2016), anti-inflammatory (Mujumdar et al., 1990), anti-asthma (Kim and Lee, 2009), anti-thyroid (Panda and Kar, 2003), and anti-tumor activities (Samykutty et al., 2013).

Previous studies have shown that PIP was capable of inhibiting spermatogenesis of male adult albino rats after 30 days of treatment (Malini et al., 1999; D’Cruz et al., 2008). In order to examine whether PIP affected spermatogenesis by interfering with Leydig cell testosterone production, in the current study we evaluated the effects of PIP on pubertal Leydig cell development. Adult Leydig cell development in rats can be conceptually divided into four stages: stem (during the whole lifespan), progenitor (at postnatal day 21), immature (between postnatal days 28 and 35), and adult Leydig cells (after postnatal day 56) (Ye et al., 2017). Late-stage pubertal development of Leydig cells is mainly involved in the transition of immature Leydig cells to adult Leydig cells. In addition to expressing all the necessary steroidogenic proteins to synthesize testosterone, immature Leydig cells also express high levels of testosterone-metabolizing enzymes—steroid 5α-reductase 1 (SRD5A1, encoded by *Srd5a1*) and AKR1C14 [3α-hydroxysteroid dehydrogenase (AKR1C14, encoded by *Akr1c14*)]. So the transition of immature Leydig cells to adult Leydig cells involves not only the increases in all testosterone-producing proteins, but also the downregulation of the two testosterone metabolizing enzymes (SRD5A1 and AKR1C14); so by adulthood, Leydig cells no longer produce dihydrotestosterone (DHT) or 5α-androstanediol (DIOL), but testosterone as the primary androgen (Ge and Hardy, 1998). In addition to the two testosterone-metabolizing enzymes, Leydig cell maturation also involves the increases of a glucocorticoid-metabolizing enzyme, 11β-hydroxysteroid dehydrogenase 1 (HSD11B1, encoded by *Hsd11b1*). The enzyme is specifically located in immature and mature Leydig cells by day 28 and later (Phillips et al., 1989), which may also represent the maturity of Leydig cells.

Leydig cell steroidogenesis and development is primarily regulated by pituitary luteinizing hormone (LH). The hormone binds to its receptor (LHCGR, encoded by *Lhcgr*) on the Leydig cell membrane, which can initiate a cAMP/PKA signaling cascade. This signaling is necessary for both the acute and trophic regulations of steroidogenesis (Ye et al., 2017). The androgen biosynthesis requires cholesterol transportation into Leydig cells by scavenger receptor class B member 1 (SCARB1, encoded by *Scarb1*) that binds to high-density lipoprotein cholesterol and transports the substrate into the Leydig cells. Cholesterol is then transported from cytosol into the inner membrane of the mitochondria by steroidogenic acute regulatory protein (STAR, encoded by *Star*). This process is considered to be the rate-limiting step in steroidogenesis. Cholesterol is then converted into pregnenolone by the cholesterol side chain cleavage enzyme (CYP11A1, encoded by *Cyp11a1*) in the inner membrane of the mitochondria and further into testosterone by a series of enzymes in smooth endoplasmic reticulum, including 3β-hydroxysteroid dehydrogenase 1 (HSD3B1, encoded by *Hsd3b1*), 17α-hydroxylase/17, 20-lyase (CYP17A1, encoded by *Cyp17a1*), and 17β-hydroxysteroid dehydrogenase 3 (HSD17B3, encoded by *Hsd17b3*). Spermatogenesis is regulated by Sertoli cells, whose function is controlled by pituitary-secreted follicle-stimulating hormone (FSH) (O’Shaughnessy et al., 2012). FSH binds to the FSH receptor in the Sertoli cell membrane to regulate its function (O’Shaughnessy et al., 2012). This study examined the effects of PIP on pubertal development of Leydig cells and the expressions and functions of multiple steroidogenic proteins along the steroidogenic pathway as well as spermatogenesis.

## Materials and Methods

### Chemicals and Animals

PIP, 8bromo-cAMP (8BR), and Percoll were purchased from Sigma (St. Louis, MO, United States). DMEM and F12 medium were purchased from Gibco (Grand Island, NY, United States). LH was obtained from NIH (United States). 22R-hydroxycholesterol (22R), pregnenolone, progesterone, androstenedione, testosterone, and DHT were purchased from Steraloids (Wilton, NH, United States). [1,2,6,7-*N*-^3^H]Testosterone and 5 -[9,11-*N*-_3_H]androstane-3,17-diol were purchased from DuPont-New England Nuclear (Boston, MA, United States). Immunohistochemical staining kit (Vectastain Elite, ABC kit, PK-6101) was purchased from Vector Laboratories (Burlingame, CA, United States). The antibodies for DIOL in radioimmunoassay (RIA) were provided by Dr. D. T. Armstrong (Department of Obstetrics and Gynecology, University of Western Ontario, London, ON, Canada). SYBR Green qPCR Kit and BCA Protein Assay Kit were purchased from Takara (Otsu, Japan). Trizol Kit was purchased from Invitrogen (Carlsbad, CA, United States). Immulite2000 Total Testosterone Kit was purchased from Sinopharm Group Medical Supply Chain Services, Co. (Hangzhou, Zhejiang, China). Radio immunoprecipitation Assay (RIPA) buffer was obtained from Bocai Biotechnology (Shanghai, China). The manufacturers of antibodies are listed in **Supplementary Table S1**. Male Sprague-Dawley rats (28 days of age) were purchased from Shanghai Animal Center (Shanghai, China). The animal study (protocol number: wydw2016-0311) was approved by the Institutional Animal Care and Use Committee of Wenzhou Medical University and was performed in accordance with the Guide for the Care and Use of Laboratory Animals.

### *In Vivo* Treatment of Rats With PIP

Eighteen 28-day-old male Sprague-Dawley rats were maintained in a 12 h dark/light cycle at temperature of 23 ± 2°C and with relative humidity of 45–55%. Water and food were provided *ad libitum*. Animals were adjusted for a week before they were randomly divided into three groups: control (0 mg/kg/day, *n* = 6), low dose of PIP (5 mg/kg/day, *n* = 6), and high dose of PIP (10 mg/kg/day, *n* = 6). PIP was suspended in normal saline. Rats were gavaged with 0, 5, or 10 mg/kg/day of PIP for 30 days. Body weights were recorded every 3 days. Rats were sacrificed at day 65 postpartum by asphyxiation with CO_2_. Trunk blood was collected in a gel glass tube and centrifuged at 1500 × *g* for 10 min to collect serum samples. Serum samples were stored at -80°C until hormone (testosterone, LH, and FSH) analysis. One testis of each animal was frozen in the liquid nitrogen and stored at -80°C for subsequent gene and protein-expression analysis. The contralateral testis was fixed in Bouin’s solution for 24 h for subsequent immunohistochemical analysis.

### Serum Hormone Assays

Serum testosterone concentration was measured by a chemiluminescence kit according to the manufacturer’s instruction (Siemens, Germany). The minimal detection limit of testosterone was 0.2 ng/ml.

Serum LH and FSH levels were detected with ELISA kits according to the manufacturer’s instruction (Chemicon, Temecula, CA, United States). Briefly, an aliquot (200 μl) of sample and 50 μl assay diluent was added to pre-coated 96-well plates. The plates were incubated at room temperature for 2 h, and washed five times with washing buffer. 100 μl peroxidase-conjugated IgG anti-LH or anti-FSH solution was added into each well at room temperature for 2 h. Then, plates were washed five times. Finally, 100 μl substrate buffer was pipetted into each well, and incubated in a dark place at room temperature for 30 min. The enzyme reaction was stopped by a stop solution. The quantification of LH or FSH level was obtained by a microplate reader at 550 nm with correction wavelength at 450 nm.

### Immunohistochemical Staining of the Testis

One testis from each rat was used for immunohistochemical staining according to the manufacturer’s instruction. Six testes per group were embedded in paraffin as a tissue array as described before (Wu et al., 2017). The immunostaining process of the tissue sections (6 μm) started with an antigen retrieval with microwave irradiation in 10 mM (pH 6.0) of citrate buffer for 10 min, followed by the blockade of endogenous peroxidase with 0.5% of H_2_O_2_ in methanol for 30 min. Sections were then incubated with CYP11A1 (biomarker of Leydig cells) or HSD11B1 (biomarkers for immature and mature Leydig cells) polyclonal antibody diluted 1:200 overnight. Diaminobenzidine was used for visualizing the antibody-antigen complexes, positively labeling Leydig cells by brown cytoplasmic staining. Mayer hematoxylin was applied as counterstaining. Non-immunized rabbit IgG was used for negative control. The Leydig cell number after CYP11A1 or HSD11B1 staining was counted. CYP11A1 and HSD11B1 densities in the individual Leydig cells were calculated. The spermatogenesis was also evaluated after Mayer hematoxylin staining.

### Counting Leydig Cell Number in the Testis

The numbers of CYP11A1-positive immature Leydig cells and HSD11B1-positive adult Leydig cells were evaluated according to a fractionator technique as previously described (Mendis-Handagama et al., 1989). Each testis was cut in eight disks, among which two were randomly selected. The selected disks were cut in four pieces each and one piece was randomly selected from the total 8. These pieces of testis were embedded in paraffin in a tissue array as described above. Ten sections were randomly sampled from each testis per rat. Sections were used for immunohistochemical staining of CYP11A1 or HSD11B1. Using a digital camera, under a 10× objective, and starting at a fixed point of the “upper” sections, the total microscopic fields per section were counted. The total number of Leydig cells was calculated by multiplying the number of Leydig cells counted in a known fraction of the testis by the inverse of the sampling probability.

### Histochemical Staining of the Epididymis

One epididymis from each rat was used for histochemical staining. Cryostat cross section (10 μm) was prepared and stained with hematoxylin and eosin stain. The sperms in the tubules of the epididymis were examined and evaluated.

### RNA Isolation and Real-Time PCR (RT-qPCR)

Total RNAs were extracted from the testes tissue with Trizol Kit according to the manufacturer’s instructions. The first strand (cDNA) was reverse-transcribed and used as the template for qPCR analysis as previously described (Li et al., 2014). The expression levels of Leydig cell genes (*Lhcgr*, *Scarb1*, *Star*, *Cyp11a1*, *Hsd3b1*, *Cyp17a1*, *Hsd17b3*, *Hsd11b1*, and *Nr5a1*) were measured using a SYBR Green qPCR Kit. The gene name and primer sequences were listed in **Supplementary Table S2**. The Ct value was read and the expression level of a target gene was calculated using a standard curve method as previously described (Li et al., 2014). The mRNA levels of all genes were adjusted to *Rps16*, a house-keeping gene as internal control.

### Western Blot Analysis

Western blot analysis of steroidogenic proteins with the total testis was performed as described (Wu et al., 2017). In brief, the testis was homogenized and boiled in equal volumes of sample loading buffer, a Tris-HCL buffer (pH 6.8) which contained 20% glycerol, 5% sodium dodecyl sulfate, 3.1% dithiothreitol, and 0.001% bromophenol blue. Samples (50 μg protein for each sample) were electrophoresed on 10% polyacrylamide gels containing sodium dodecyl sulfate. After electrophoresis, the proteins were transferred onto a nitrocellulose membrane, and the membrane was incubated with 5% non-fat milk for 1 h to block the non-specific binding. Then, the membrane was incubated with primary antibodies against the following antigens: LHCGR, SACRB1, STAR, CYP11A1, HSD3B1, CYP17A1, HSD11B1, NR5A1 and pAKT1, pAKT2, AKT1, AKT2, pERK1/2, ERK1/2, as well as ACTB (listed in **Supplementary Table S1**). The membranes were washed and incubated with a 1:5000 dilution of goat anti-rabbit antiserum that was conjugated to horseradish peroxidase. The washing step was repeated three times, and immunoreactive bands were visualized by chemiluminescence using an ECL kit (Amersham, Arlington Heights, IL, United States). The intensity of each protein band was quantified using Image J software. The Leydig and Sertoli cell proteins were adjusted to a house-keeping protein ACTB, as an internal control.

### Immature Leydig Cell Isolation

Eighteen 35-day-old male Sprague-Dawley rats were sacrificed by asphyxiation with CO_2_. Testes were removed and immature Leydig cells were purified as described previously (Ge and Hardy, 1998). In brief, testes were perfused with a M199 solution containing 0.1 mg/ml collagenase via the testicular artery, digested with a mixture of 0.25 mg/ml collagenase and 0.25 mg/ml DNase for 15 min. After filtering with 100 μm nylon mesh, the cells were separated with Percoll gradient as previously described (Ge and Hardy, 1998). The cells with density of 1.070–1.088 g/ml were collected and washed. The purities of Leydig cell fractions were evaluated by histochemical staining for HSD3B1 (a biomarker of Leydig cells) with 0.4 mM etiocholanolone as the steroid substrate and NAD^+^ as a cofactor as described (Payne et al., 1980). The purities of immature Leydig cells were >95%.

### Treatment of Leydig Cell *in Vitro*

Immature Leydig cells were seeded into the 6-well culture plates with 10^5^ cells per well. Leydig cells were treated with 50 μM PIP in 2.0 mL of DMEM: F12 medium for 24 h. Because immature Leydig cells produced about 80% DIOL and 10% testosterone (Ge and Hardy, 1998), media were harvested for the measurement of both testosterone and DIOL concentrations. To further dissect the action site(s) of PIP on androgen biosynthesis and metabolism, we isolated immature Leydig cells and treated the cells with 50 μM PIP, in the presence of stimulating hormone (LH, 10 ng/ml), signaling compound (8Br-cAMP, 8BR, 10 mM) and steroidogenic enzyme substrates, including CYP11A1 (22R-hydroxycholesterol, 22R, 5 μM), HSD3B1 (pregnenolone, P5, 5 μM), CYP17A1 (progesterone, P4, 5 μM), HSD17B3 (androstenedione, D4, 5 μM), and SRD5A1 (testosterone, T, 5 μM). The treatments lasted for 3 h and the medium DIOL and testosterone were measured. The substrate information is listed in **Supplementary Table S2**. The treatments were carried out on a 24-well culture plate that contained 0.05 × 10^6^ cells/well.

### Measurement of DIOL and Testosterone Levels in Medium by RIA

5α-Androstanediol and testosterone concentrations in the medium were assayed with the tritium-based RIA as described previously (Ge and Hardy, 1998) using the commercial RIA kits (IBL, United States) with DIOL or testosterone antibody. The minimum detectable concentration of the assay for DIOL and testosterone was 5 pg/ml. The internal control contained 100 pg/ml DIOL or testosterone. Interassay and intraassay variations of DIOL and testosterone were within 15%.

### Statistical Analysis

Data were expressed as the mean ± SEM. *P* < 0.05 was considered statistically significant. The differences of groups were evaluated by one-way ANOVA followed by *ad hoc* Dunnett’s multiple comparisons test to compare with the control. The data of Western blot bands were analyzed by a paired Student’s *t*-test followed by Sidak adjustment to compare the difference with the control.

## Results

### General Effects

To analyze the general effects of PIP, body weights were recorded before or after oral administration of PIP from postnatal day 35 to day 65 (**Table 1**). Testis weights were recorded at the end of PIP treatment. PIP did not affect rat body weights during the course of treatment. PIP did not affect rat testis weights at the end of PIP treatment either. No mortalities and abnormal activities were observed in the rats of any group.

**Table 1:** Body weight and testis weight after treatment of piperine.

Parameters	Dosage (mg/kg)		
	0	5	10
Body weight			
Before piperine	120.50 ± 5.20	115.00 ± 8.64	125.00 ± 7.98
After piperine treatment (days)			
7	167.5 ± 9.29	165.00 ± 11.22	178.8 ± 9.78
14	219.50 ± 14.66	211.25 ± 13.5	226.00 ± 10.07
21	255.00 ± 15.75	253.5 ± 13.5	262.60 ± 13.33
28	273.50 ± 17.00	273.75 ± 13.70	291.2 ± 16.59

Testis weight	2.82 ± 0.29	2.84 ± 0.20	2.75 ± 0.22

*Mean ± SEM, *n* = 6*.

### PIP Increases Serum Testosterone Levels *in Vivo*

**FIGURE 1:**
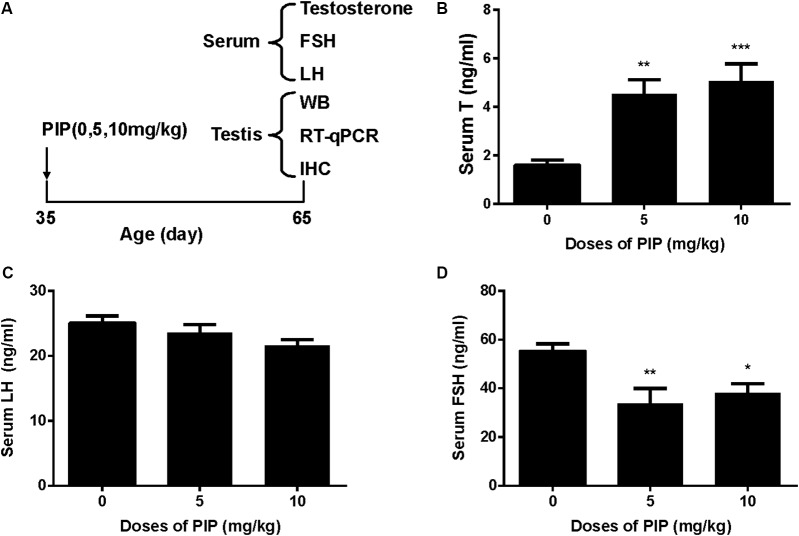
Regimen of piperine (PIP) treatment and its effects on serum hormone levels. **(A)** PIP regimen; **(B)** serum testosterone (T); **(C)** serum luteinizing hormone (LH); **(D)** serum follicle-stimulating hormone (FSH). Mean ± SEM, *n* = 6. ^∗^*P* < 0.05, ^∗∗^*P* < 0.01, ^∗∗∗^*P* < 0.001 indicate significant differences when compared to the control (0 mg/kg), respectively.

**FIGURE 2:**
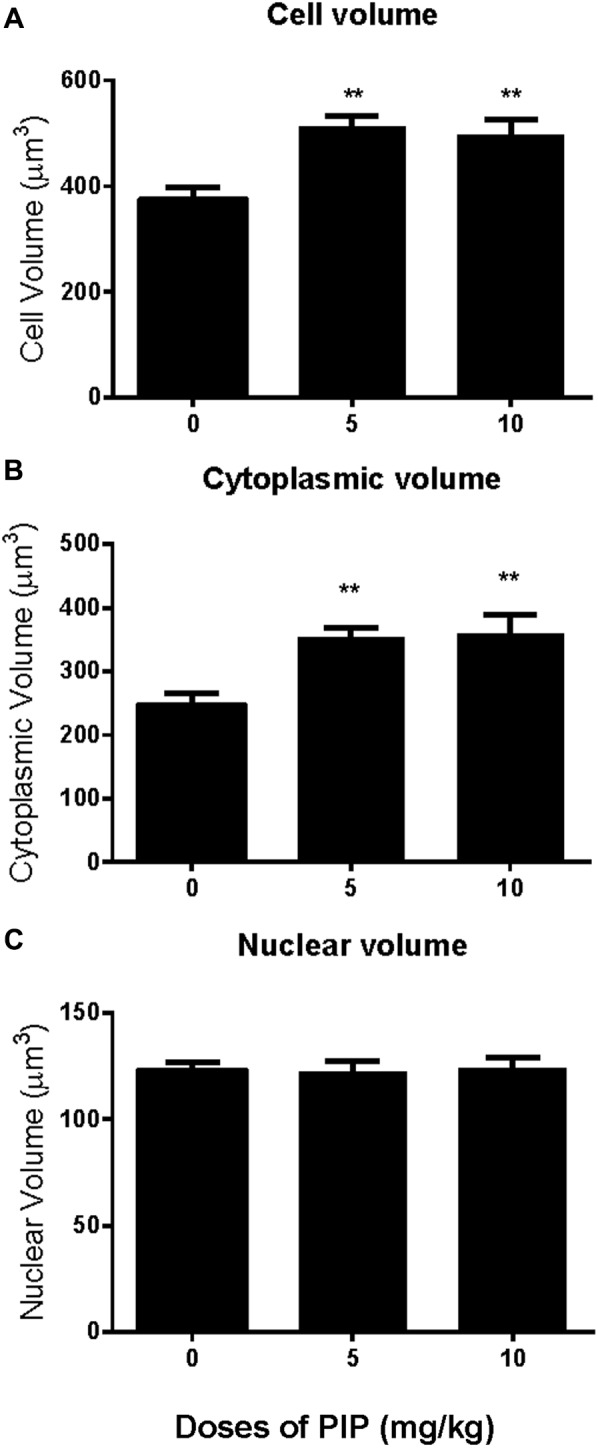
Effects of PIP treatment on Leydig cell volume, nuclear volume, and cytoplasmic volume. **(A)** Leydig cell volume; **(B)** Leydig cell cytoplasmic volume; **(C)** Leydig cell nuclear volume. Mean ± SEM, *n* = 6. ^∗∗^*P* < 0.01 indicates significant difference when compared to the control (0 mg/kg). Scale bars = 50 μm.

Rats were gavaged PIP (0, 5, 10 mg/kg) from postnatal day 35 to day 65 (**Figure 1A**). Sera were collected at age of 65 days for hormone (testosterone, LH, and FSH) analysis (**Figures 1B–D**). When compared with control (0 mg/kg PIP), PIP dose-dependently increased testosterone levels by 5 and 10 mg/kg doses (**Figure 1B**). This result suggested that PIP might promote Leydig cell development. Further analysis of Leydig cell population indicated that PIP treatment increased Leydig cell size and cytoplasmic size without affecting the nuclear size (**Figures 2A–C**). This indicated that PIP might stimulate Leydig cell maturation process, since the more mature the cell is, the larger the cell is. To examine whether PIP affected Leydig cell development by acting on hypothalamic-pituitary level, we measured serum LH and FSH levels (**Figures 1C,D**). Interestingly, PIP did not affect LH levels but reduced serum FSH levels.

### PIP Increases Leydig Cell Number *in Vivo*

**FIGURE 3:**
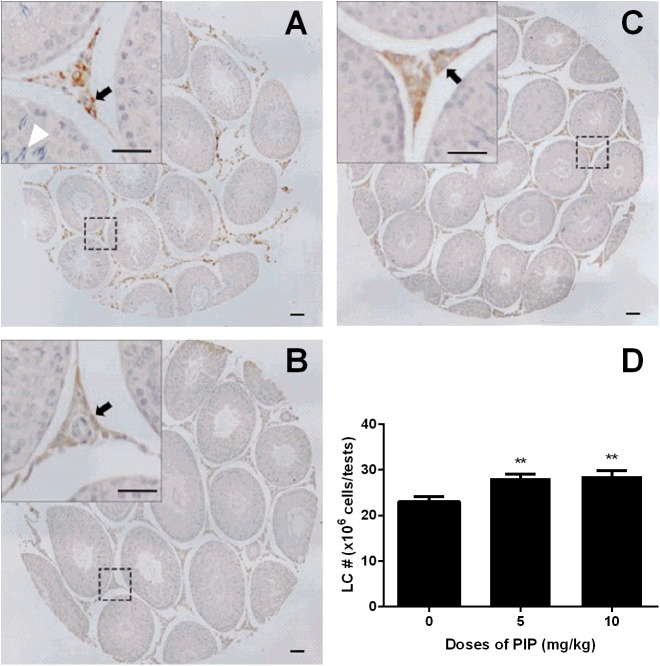
Effects of PIP treatment on CYP11A1 expression cells. **(A)** Control; **(B)** 5 mg/kg PIP; **(C)** 10 mg/kg PIP. Arrows points to the Leydig cells. Insert is the magnification of the square image. **(D)** Quantification of CYP11A1 positive cell numbers. Mean ± SEM, *n* = 6. ^∗∗^*P* < 0.01 indicates a significant difference when compared to the control (0 mg/kg).

CYP11A1, a universal biomarker of all Leydig cells in this lineage (Ye et al., 2017), was used to identify all developmental stages of Leydig cells. Cells with brown cytosolic staining in the interstitium were identified as Leydig cells. We counted the CYP11A1-positive cells (**Figures 3A–D**) and found that PIP dose-dependently increased the total number of Leydig cells (**Figure 3D**). This indicated that PIP promotes Leydig cell development from the CYP11A1-negative stem cells during puberty. To evaluate the numbers of Leydig cells at the advanced stages, we performed HSD11B1 staining for Leydig cells. The number of HSD11B1-positive Leydig cells showed a similar increase in PIP-treated animals as CYP11A1-positive cells (**Supplementary Figure S1**), suggesting that this food supplement consistently increased the overall number of Leydig cells without affecting specific developmental stages.

### PIP Delays Spermatogenesis *in Vivo*

Since PIP significantly reduced serum FSH levels, we wanted to examine whether such a change might have adverse effects on spermatogenesis. Morphological analysis of the testis sections indicated that PIP indeed dose-dependently inhibited spermatogenesis. As showed in **Figure 3A**, the seminiferous tubules of the animals in the control group contained abundant spermatocytes, which were affected significantly by PIP treatment. As shown in **Figure 3C**, animals with 10 mg/kg PIP treatment had seminiferous tubules that contained very few spermatocytes. These effects on the testis were also confirmed by the changes in sperm counts in the epididymis. As shown in **Supplementary Figure S2**, the control epididymal tubules contained a full load of sperms with normal morphology. With 5 mg/kg PIP treatment, the sperm count was apparently reduced significantly. With 10 mg/kg PIP treatment, sperm count was reduced further, with sperms being aggregated together. The morphological results of both testis and epididymis indicated clearly that PIP blocked spermatogenesis maturation.

### PIP Increases Leydig Cell-Specific mRNA Levels *in Vivo*

**FIGURE 4:**
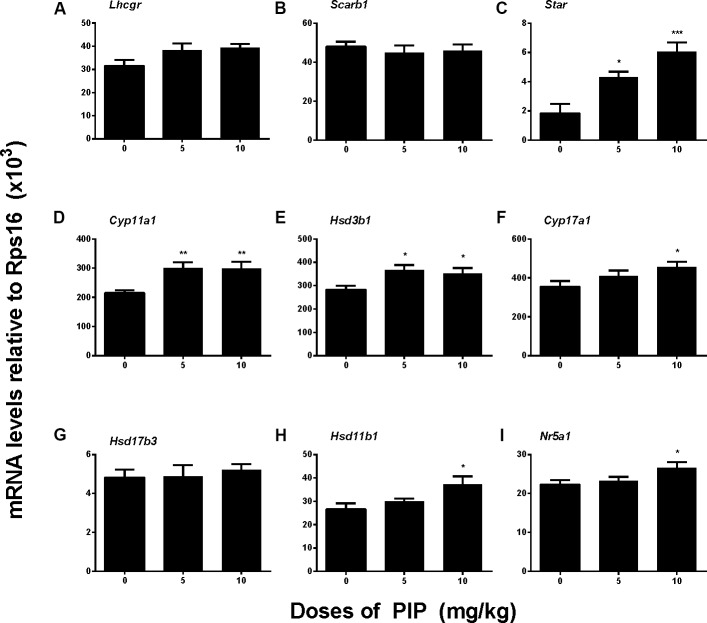
Gene expression levels in the testes of rats with or without piperine (PIP) treatment. Genes in the Leydig cell: **(A)** *Lhcgr*, **(B)** *Scarb1*, **(C)** *Star*, **(D)** *Cyp11a1*, **(E)** *Hsd3b1*, **(F)** *Cyp17a1*, **(G)** *Hsd17b3*, **(H)** *Hsd11b1*, and **(I)** *Nr5a1*. Mean ± SEM, *n* = 6. ^∗^*P* < 0.05, ^∗∗^*P* < 0.01, ^∗∗∗^*P* < 0.001 indicate significant differences when compared to the control (0 mg/kg), respectively.

To further examine whether PIP treatment might affect any of the steroidogenic gene expressions, we measured the mRNA levels of eight proteins that play roles in Leydig cell steroidogenesis, including *Lhcgr*, *Scarb1*, *Star*, *Cyp11a1*, *Hsd3b1*, *Cyp17a1*, *Hsd11b1*, and *Nr5a1*. *In vivo* PIP treatment increased expression levels of *Star*, *Cyp11a1*, and *Hsd3b1* by 5 mg/kg and above and also increased *Cyp17a1*, *Hsd11b1*, and *Nr5a1* levels by 10 mg/kg. However, the treatment did not affect *Hsd17b3* by any doses (**Figure 4**). This suggested that PIP treatment was indeed able to upregulate the expression levels of multiple steroidogenic genes, but the effects might not be equal across all the genes.

### PIP Increases Leydig Cell-Specific Protein Levels *in Vivo*

**FIGURE 5:**
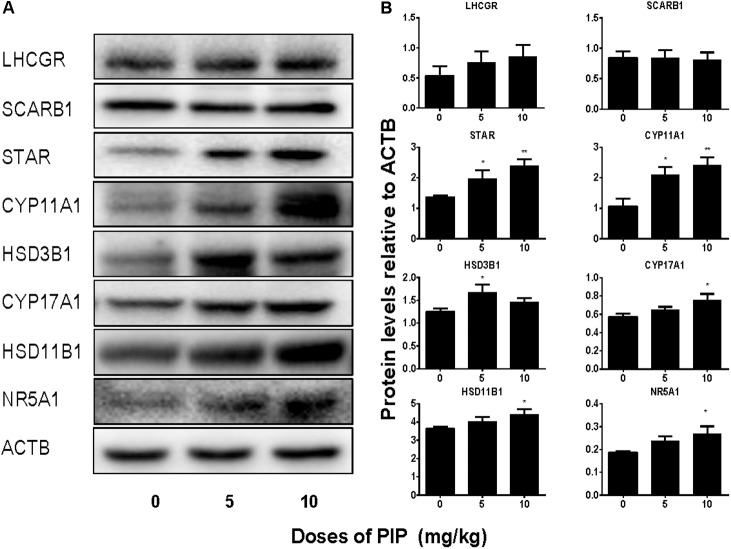
Protein levels of the testes of rats with or without piperine (PIP) treatment. Protein expressions in the testis: **(A)** Western blot band. **(B)** Quantification of protein levels. Mean ± SEM, *n* = 6. ^∗^*P* < 0.05, ^∗∗^*P* < 0.01, indicate significant differences when compared to the control (0 mg/kg), respectively.

**FIGURE 6:**
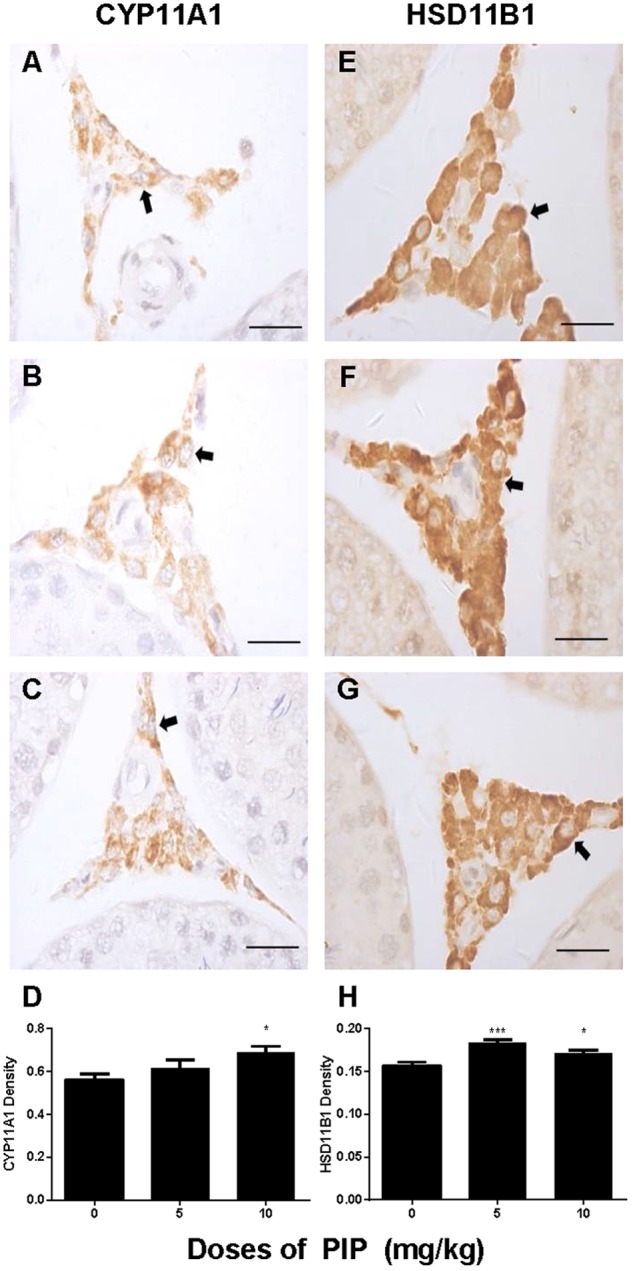
Semi-quantitative analysis of CYP11A1 and HSD11B1 positive-cells after piperine (PIP) treatment. Immunohistochemical images (1) CYP11A1: **(A–C)** and (2) HSD11B1: **(E–G)**. Arrow points to the Leydig cell. **(D,H)** Quantification of CYP11A1 and HSD11B1 density. Mean ± SEM, *n* = 6. ^∗^*P* < 0.05, ^∗∗^*P* < 0.01, ^∗∗∗^*P* < 0.001 indicate a significant difference when compared to the control (0 mg/kg), respectively. Scale bar = 50 μm.

We also measured the levels of Leydig cell steroidogenic proteins (LHCGR, SCARB1, STAR, CYP11A1, HSD3B1, CYP17A1, HSD11B1, and NR5A1) in the testis after PIP treatment. In general, the changes in these protein levels were in parallel with changes in their mRNA levels (**Figure 5**). We further measured CYP11A1 and HSD11B1 protein levels of individual Leydig cells using the immunohistochemical staining of testis sections (tissue-array) (**Figure 6**). PIP treatment increased CYP11A1 density by 10 mg/kg and increased HSD11B1 density by 5 mg/kg and above. Overall, these results further confirmed that PIP promoted the development of Leydig cells during puberty.

**FIGURE 7:**
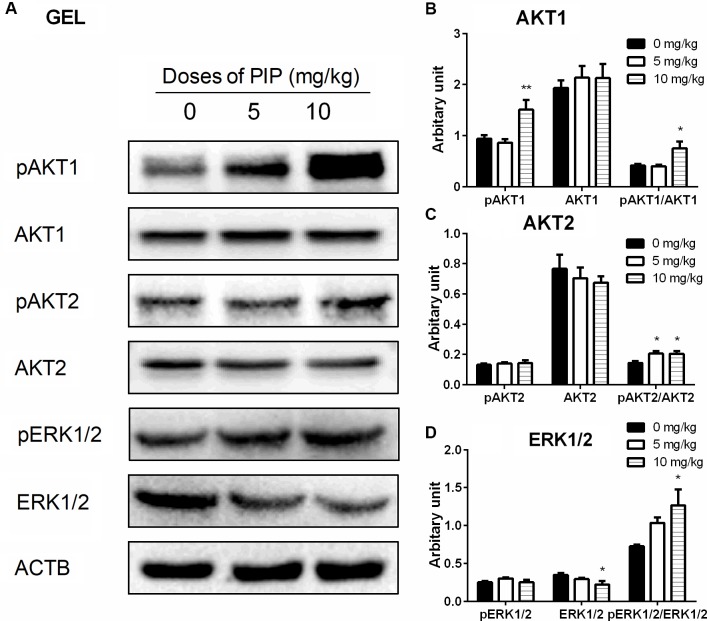
The kinase and phosphorylated kinase protein levels of the testes of rats with or without piperine (PIP) treatment. **(A)** Western blot band. **(B–D)** Quantification of kinase and phosphorylated kinase protein levels. Mean ± SEM, *n* = 6. ^∗^*P* < 0.05, ^∗∗^*P* < 0.01 indicate significant differences when compared to the control (0 mg/kg), respectively.

Many studies have demonstrated that ERK1/2 and AKT pathways participated in development of Leydig cells (Manna et al., 2006, 2007; Shiraishi and Ascoli, 2007). Herein, we investigated the downstream signals after PIP treatment in the testis. PIP significantly increased the ratio of phospho-AKT1 (pAKT1)/AKT1, phosphos-AKT2 (pAKT2)/AKT2, and phospho-ERK1/2 (pERK1/2)/ERK1/2 in the PIP-treated testis (**Figure 7**). These results indicated that ERK1/2 and AKT pathways are involved in the PIP-mediated stimulation of Leydig cell development.

### PIP Directly Stimulates Androgen Production *in Vitro*

**FIGURE 8:**
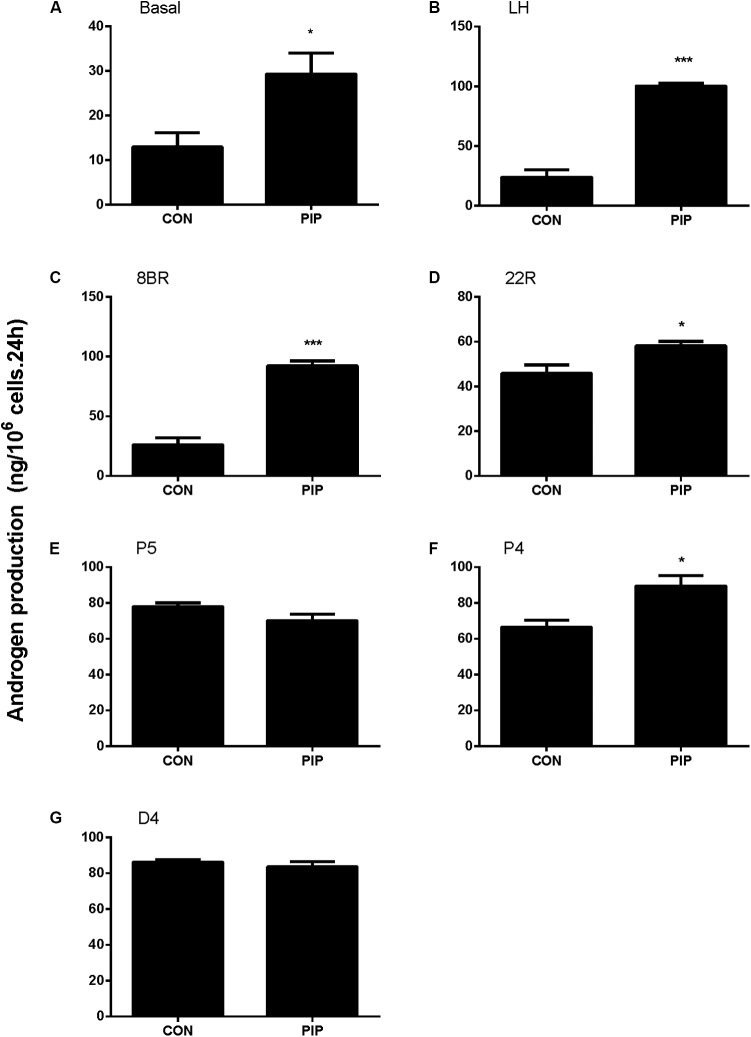
Concentration-dependent effects of piperine (PIP) on basal androgen productions of Leydig cells. Rat immature Leydig cells were cultured with 0.5–50 μM PIP for 24 h. The levels of testosterone and DIOL were measured. **(A–G)** Androgen levels without (**A**, basal) or with addition of different agents **(B–G)** from LH, 8BR, 22R, P5, P4, and D4, respectively. Mean ± SE, *n* = 6. ^∗^*P* < 0.05, ^∗∗∗^*P* < 0.001 indicates significant difference when compared to the control (0 μM), respectively.

To examine whether PIP may have direct effects on Leydig cell steroidogenesis, we have treated immature Leydig cells isolated from 35-day-old rats with 50 μM PIP in the absence (basal) or presence of LH (10 ng/ml) or 8BR (10 mM) for 24 h. PIP significantly increased basal, LH-stimulated, and 8BR-stimulated total androgen production (**Figures 8A–C**). In order to dissect the potentially acting site(s) of PIP on the androgen biosynthetic enzymes (CYP11A1, HSD3B1, CYP17A1, and HSD17B3), 5 μM of substrate for each enzyme (22R for CYP11A1, P5 for HSD3B1, P4 for CYP17A1, D4 for HSD17B3) were co-incubated with 50 μM PIP. The names and abbreviation of substrates are listed in **Supplementary Table S3**. PIP increased 22R- and P4-stimulated androgen productions, indicating that PIP may increase CYP11A1 and CYP17A1 activities (**Figures 8D–G**). Because DIOL and testosterone accounts for about 80 and 10% androgens produced by immature Leydig cells (Ge and Hardy, 1998) and DIOL is the metabolite of testosterone after SRD5A1 and AKR1C14 catalysis, we further analyzed the DIOL and testosterone productions separately (**Table 2**). We found that testosterone (T)-stimulated DIOL level was not changed by PIP, indicating that PIP did not affect androgen-metabolizing enzyme activities.

**Table 2:** The androstanediol (DIOL) and testosterone production in rat immature Leydig cells after piperine treatment.

	DIOL (ng/10^6^ cells 24 h)		Testosterone (ng/10^6^ cells 24 h)	
	Control	Piperine	Control	Piperine
Basal	10.00 ± 3.66	26.92 ± 6.56^∗∗^	2.31 ± 1.41	3.22 ± 0.61
LH	16.63 ± 7.67	70.23 ± 5.49^∗∗∗^	7.31 ± 4.73	30.04 ± 5.51^∗∗∗^
8BR	17.68 ± 3.63	62.62 ± 11.74^∗∗∗^	8.54 ± 29.57	29.57 ± 4.70^∗∗∗^
22R	25.48 ± 2.41	25.74 ± 2.80	20.36 ± 7.24	32.43 ± 1.92^∗^
P5	41.09 ± 3.55	29.22 ± 7.79^∗^	36.68 ± 2.02	40.85 ± 3.83
P4	33.75 ± 5.22	53.37 ± 6.70^∗∗^	32.73 ± 6.42	36.08 ± 6.23
D4	49.77 ± 3.03	50.32 ± 4.25	36.30 ± 2.38	33.12 ± 3.35
T	57.22 ± 7.88	53.42 ± 2.37		

*Mean ± SEM, *n* = 6. ^∗^*P* < 0.05, ^∗∗^*P* < 0.01, ^∗∗∗^*P* < 0.001 indicates significant difference when compare to the control. LH, luteinizing hormone; 8BR, 8bromo-cAMP; 22R, 22R-OH-cholesterol; P5, pregnenolone; P4, progesterone; D4, androstenedione; T, testosterone*.

## Discussion

In the present study, we demonstrated that PIP significantly promoted Leydig cell development during puberty by increasing Leydig cell numbers, Leydig cell size, and the expression levels of steroidogenesis-related proteins. Further *in vitro* study also identified that PIP was able to increase androgen productions of immature Leydig cells by increasing CYP11A1 and CYP17A1 activities. However, PIP may have adverse effects on spermatogenesis, possibly via reducing FSH action.

The present study showed that PIP had stimulatory effects on androgen production if given to animals during puberty. PIP increased androgen production by several mechanisms: increasing Leydig cell number (**Figure 3**), elevating the expression of key biosynthetic enzymes such as CYP11A1 in the invidual Leydig cell (**Figure 6**), and promoting the maturity of the Leydig cell such as increasing Leydig cell size and cytoplasmic size (**Figure 2**), and increasing HSD11B1 expression (**Figure 6**). These effects may not be mediated via pituitary-secreted LH, since serum LH level was unchanged (**Figure 1C**). A direct effect of PIP may be involved. Indeed, PIP was able to stimulate androgen production of immature Leydig cells even if it was incubated with the cells in a short period (**Figure 8** and **Table 2**). This direct effect may be mainly mediated by increasing CYP11A1 and CYP17A1 activities (**Figure 7**). The exact mechanism of PIP-mediated promotion of Leydig cell deveopment is still unclear. Interestingly, the effects of PIP on rat Leydig cell development during puberty are different from what were observed for its effects on testis in adult rats, which had lower intratesticular testosterone levels and elevated LH and FSH levels (Malini et al., 1999). The reason for this difference is still unclear. We speculated that this might be contributed to by the age difference. Indeed, the serum FSH levels in the pubertal rats were significantly lower than in the control in the current study (**Figure 1D**).

Several studies showed that ERK1/2 and AKT signaling pathways participated in Leydig cell development (Manna et al., 2006, 2007; Renlund et al., 2006; Shiraishi and Ascoli, 2007). There are several factors such as epidermal growth factor, insulin-like growth factor 1, and LH to regulate ERK1/2 or AKT. The epidermal growth factor, after binding to its receptor, caused activation of ERK1/2 in Leydig cells (Hirakawa and Ascoli, 2003; Shiraishi and Ascoli, 2007, 2008) and the epidermal growth-factor receptor is the partial mediator of the LHCGR-provoked activation of ERK1/2 cascade in immature Leydig cells (Hirakawa and Ascoli, 2003; Shiraishi and Ascoli, 2007, 2008). StAR has been shown to be the substrate of ERK1/2 and phosphorylaton of ERK1/2 has been shown to participate in cholesterol transportation (Poderoso et al., 2008). Apparently, although the serum LH levels were not altered after PIP treatment, the significant increase of LHCGR could lead to the activation of ERK1/2 as shown in the present study (**Figure 7**). AKT is also a key regulator of Leydig cell development. AKT is mainly regulated by insulin-like growth factor 1 (Tai et al., 2009) and kit ligand (Rothschild et al., 2003). Insulin-like growth factor 1 knockout in mice showed a reduced expression of several Leydig cell biomarkers such as *Star*, *Cyp11a1*, *Hsd3b1*, and *Cyp17a1*, Leydig cell hypoplasia, and lowered testosterone synthesis (Baker et al., 1996; Hu et al., 2010). Blockade of kit ligand signaling also led to the reduced testosterone production in Leydig cells (Rothschild et al., 2003). AKT phosphorylation relies on the activation of phosphatidylinositol 3-kinase (Tai et al., 2009; Zhou et al., 2016). Phosphatidylinositol 3-kinase is activated by insulin-like growth factor 1 and kit ligand (Rothschild et al., 2003). Although we have not performed a complete characterization of the pathways by which the PIP enhances ERK1/2 and AKT phosphorylation, the present data presented show that ERK1/2 and AKT pathways are associated with Leydig cell development.

NR5A1 may also be invovled in PIP-mediated action. PIP *in vivo* significantly increased NR5A1 mRNA and protein levels (**Figures 4**, **5**). NR5A1 is a ligand-free nuclear receptor and is a critical transcription factor for promoting Leydig cell development. NR5A1 can bind to the promoters of many Leydig cell-specific genes such as *Star*, *Cyp11a1*, *Cyp17a1*, and *Hsd3b1* (Sandhoff et al., 1998; Chen et al., 1999; Hu et al., 2001; Schimmer et al., 2011). Null mutation of NR5A1 caused gonadal agenesis (Sadovsky et al., 1995). Forced expression of NR5A1 can even convert stem cells or fibroblasts into steroidogenic cells by transcriptionally promoting the expression of LHCGR and other steroidogenic enzymes (CYP11A1, HSD11B1, CYP17A1, and HSD17B3) (Yang et al., 2017).

Previous studies have shown that PIP inhibited spermatogenesis on male adult albino rats after 30 days of treatment (Malini et al., 1999; D’Cruz et al., 2008). In the present study, we extended these observations by showing that PIP was also able to delay the initiation of spermatogenesis if given during puberty. Our results indicated that PIP treatments dose-dependently reduced the numbers of developing germ cells in the testis (**Figure 3**) and the sperm numbers in the epididymis (**Supplementary Figure S2**). The mechanism by which PIP suppresses spermatogenesis during the puberty is still unclear. The drops in serum FSH may be involved. It has been shown previously that the initiation of rat spermatogenesis required FSH while the maintenance of the process did not (Zirkin et al., 1994). Further evidence that PIP may affect spermatogenesis with different mechanisms during puberty and at adulthood comes from the fact that FSH level was actually increased in adult rats after 10 mg/kg PIP treatment (Chinta et al., 2017).

The doses of PIP used in the rats were 5 and 10 mg/kg which were adopted from another study for adult rats (Malini et al., 1999). The doses of PIP in the present study may be clinically relevant, since PIP in clinic trials has been tested for its potential to affect bioavailability of other compounds in food and dietary supplements and the dose of PIP used was 20 mg for humans (Shoba et al., 1998; Bedada and Boga, 2017).

## Conclusion

Pubertal exposure of male rats to PIP may have diverged effects on testicular development. The treatment of PIP promotes Leydig cell development and maturation but inhibits spermatogenesis. The effects on Leydig cell development may be mediated by stimulations of multiple steroidogenic gene expressions, while the effects on spermatogenesis may be mediated by its effects on pituitary FSH release. ERK1/2 and AKT pathways may be involve in the piperine-mediated stimulation of Leydig cell development.

## Author Contributions

QL and R-SG conceptualized the study design and analyzed the data. XwC, FG, JL, SB, YC, DL, YL, TH, XfC, and QZ performed the experiments and collected the data. R-SG wrote the manuscript.

## Conflict of Interest Statement

The authors declare that the research was conducted in the absence of any commercial or financial relationships that could be construed as a potential conflict of interest.

## Supplementary Material

FIGURE S1 Immunohistochemical staining of HSD11B1 in testis sections of piperine (PIP) treated rats. **(A)** Control; **(B)** 5 mg/kg PIP; **(C)** 10 mg/kg PIP. Arrow points to the Leydig cell. Insert is the magnification of the square image. **(D)** Quantification of HSD11B1 positive cell numbers. Mean ± SEM, *n* = 6. ^∗∗^*P* < 0.01 indicates a significant difference when compared to the control (0 mg/kg).

FIGURE S2 Hematoxylin staining of frozen rat epididymal sections after piperine (PIP) treatment. **(A)** Control; **(B)** 5 mg/kg PIP; **(C)** 10 mg/kg PIP. Arrows points to sperms. Bar = 50 μm.

TABLE S1 Antibodies.

TABLE S2 Primer information.

TABLE S3 Signaling and enzyme activity analysis by piperine.
