# Supplementary figures and images for: Diverged Effects of Piperine on Testicular Development: Stimulating Leydig Cell Development but Inhibiting Spermatogenesis in Rats
Source: Front Pharmacol. 2018 Mar 28;9:244. doi: 10.3389/fphar.2018.00244 (PMC5883368; doi:10.3389/fphar.2018.00244)

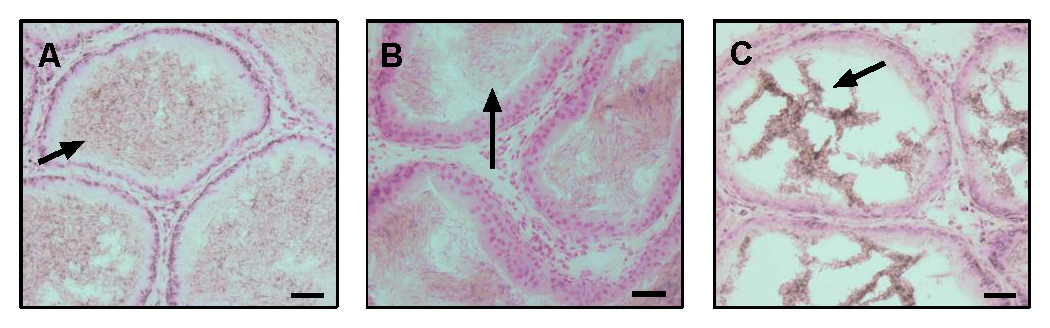

Supplement: FIGURE S2 — Hematoxylin staining of frozen rat epididymal sections after piperine (PIP) treatment. (A) Control; (B) 5 mg/kg PIP; (C) 10 mg/kg PIP. Arrows points to sperms. Bar = 50 μm. [file Image_2.TIFF]
