# Supplementary material for: Diverged Effects of Piperine on Testicular Development: Stimulating Leydig Cell Development but Inhibiting Spermatogenesis in Rats
Source: Front Pharmacol. 2018 Mar 28;9:244. doi: 10.3389/fphar.2018.00244 (PMC5883368; doi:10.3389/fphar.2018.00244)
Supplement: TABLE S1 — Antibodies. [file Table_1.DOCX]

**Supplementary Table S1. Antibodies**

| **Antibody** | **Species** | **Vendor (City, State, catalogue)** | **Dilution** | |
| --- | --- | --- | --- | --- |
|  |  |  | **WB** | **HS** |
| ACTB | Rabbit | Cell Signaling Technology (Danvers, MA) | 1:1000 | ND |
| NR5A1 | Rabbit | Abcam (San Francisco, CA) | 1:1000 | ND |
| LHCGR | Rabbit | Multi Sciences (Hangzhou, China) | 1:1000 | ND |
| STAR | Rabbit | Cell Signaling Technology (Danvers, MA) | 1:1000 | ND |
| SCARB1 | Rabbit | Multi Sciences (Hangzhou, China) | 1:1000 | ND |
| CYP11A1 | Rabbit | Cell Signaling Technology (Danvers, MA) | 1:1000 | 1:200 |
| CYP17A1 | Rabbit | Abcam (San Francisco, CA) | 1:1000 | ND |
| HSD11B1 | Rabbit | Abcam (San Francisco, CA) | 1:1000 | 1: 200 |
| HSD3B1 | Rabbit | Abcam (San Francisco, CA) | 1:1000 | ND |
| AKT1 | Rabbit | Abcam (San Francisco, CA) | 1:2000 | ND |
| pAKT1 | Rabbit | Abcam (San Francisco, CA) | 1:5000 | ND |
| AKT2 | Rabbit | Abcam (San Francisco, CA) | 1:1000 | ND |
| pAKT2 | Rabbit | Abcam (San Francisco, CA) | 1:500 | ND |
| ERK1/2 | Mouse | Abcam (San Francisco, CA) | 1:1000 | ND |
| pERK1/2 | Mouse | Abcam (San Francisco, CA) | 1:10000 | ND |

ND = Not detected; WB = Western blot; HS = Histochemical staining.
