# Supplementary material for: Diverged Effects of Piperine on Testicular Development: Stimulating Leydig Cell Development but Inhibiting Spermatogenesis in Rats
Source: Front Pharmacol. 2018 Mar 28;9:244. doi: 10.3389/fphar.2018.00244 (PMC5883368; doi:10.3389/fphar.2018.00244)
Supplement: TABLE S2 — Primer information. [file Table_2.docx]

**Supplementary Table S2. Primer information**

| **Primer**  **Symbol** | **Gene name** | **Primer direction** | **Sequences (5’to 3’)** | **PCR**  **(bp)** | **Accession** |
| --- | --- | --- | --- | --- | --- |
| *Nr5a1* | Nuclear receptor  steroidogenic factor 1 | Forward | CAGAGCTGCAAAATCGACAA | 186 | NM_053344 |
|  |  | Reverse | CCCGAATCTGTGCTTTCTTC |  |  |
| *Lhcgr* | Luteinizing hormone receptor | Forward | CTGCGCTGTCCTGGCC | 103 | NM_012978 |
|  |  | Reverse | CGACCTCATTAAGTCCCCTGAA |  |  |
| *Scarb1* | Scavenger receptor class B, member 1 | Forward | ATGGTACTGCCGGGCAGAT | 117 | NM_031541 |
|  |  | Reverse | CGAACACCCTTGATTCCTGGTA |  |  |
| *Star* | Steroidogenic acute regulatory protein | Forward | CCCAAATGTCAAGGAAATCA | 187 | NM_031558 |
|  |  | Reverse | AGGCATCTCCCCAAAGTG |  |  |
| *Cyp11a1* | Cholesterol side chain cleavage enzyme | Forward | AAGTATCCGTGATGTGGG | 127 | NM_017286 |
|  |  | Reverse | TCATACAGTGTCGCCTTTTCT |  |  |
| *Hsd3b1* | 3β-Hydroxysteroid dehydrogenase 1 | Forward | CCCTGCTCTACTGGCTTGC | 189 | NM_001007719 |
|  |  | Reverse | TCTGCTTGGCTTCCTCCC |  |  |
| *Cyp17a1* | P450 17α-hydroxylase/ 17,20-lyase | Forward | TGGCTTTCCTGGTGCACAATC | 90 | NM_012753 |
|  |  | Reverse | TGAAAGTTGGTGTTCGGCTGAAG |  |  |
| *Hsd17b3* | 17β-Hydroxysteroid dehydrogenase 3 | Forward | TGAAAGTTGGTGTTCGGCTGAAG | 202 | NM_054007 |
|  |  | Reverse | TGAAAGTTGGTGTTCGGCTGAAG |  |  |
| Rps16 | Ribosomal protein s16 | Forward | AAGTCTTCGGACGCAAGAAA | 148 | NM_001169146 |
|  |  | Reverse | TTGCCCAGAAGCAGAACAG |  |  |
