# Supplementary material for: Diverged Effects of Piperine on Testicular Development: Stimulating Leydig Cell Development but Inhibiting Spermatogenesis in Rats
Source: Front Pharmacol. 2018 Mar 28;9:244. doi: 10.3389/fphar.2018.00244 (PMC5883368; doi:10.3389/fphar.2018.00244)
Supplement: TABLE S3 — Signaling and enzyme activity analysis by piperine. [file Table_3.docx]

**Supplementary Table S3.** Signaling and enzyme activity analysis by piperine

| **Compound and hormone** | | | Targets |
| --- | --- | --- | --- |
| **Full name** | **Abbreviation** | **Conc. (μM)** |  |
| Luteinizing hormone | LH | 10 ng/ml | LHCGR |
| 8bromo-cAMP | 8BR | 10 mM | cAMP/PKA signal |
| 22R-OH-cholesterol | 22R | 5 | CYP11A1 |
| Pregnenolone | P5 | 5 | HSD11B1 |
| Progesterone | P4 | 5 | CYP17A1 |
| Androstenedione | D4 | 5 | HSD17B3 |
| Testosterone | T | 5 | SRD5A1 |
